# Classification of feline hypertrophic cardiomyopathy-associated gene variants according to the American College of Medical Genetics and Genomics guidelines

**DOI:** 10.3389/fvets.2024.1327081

**Published:** 2024-02-02

**Authors:** Fréderique Boeykens, Marie Abitbol, Heidi Anderson, Tanushri Dargar, Paolo Ferrari, Philip R. Fox, Jessica J. Hayward, Jens Häggström, Stephen Davison, Mark D. Kittleson, Frank van Steenbeek, Ingrid Ljungvall, Leslie A. Lyons, Maria Longeri, Åsa Ohlsson, Luc Peelman, Caroline Dufaure de Citres, Pascale Smets, Maria Elena Turba, Bart J. G. Broeckx

**Affiliations:** ^1^Laboratory Animal Genetics, Department of Veterinary and Biosciences, Faculty of Veterinary Medicine, Ghent University, Merelbeke, Belgium; ^2^Univ Lyon, VetAgro Sup, Marcy-l’Etoile, France & Institut NeuroMyoGène INMG-PNMG, CNRS UMR5261, INSERM U1315, Faculté de Médicine, Rockefeller, Université Claude Bernard Lyon 1, Lyon, France; ^3^Wisdom Panel, Mars Petcare Science & Diagnostics, Helsinki, Finland; ^4^Osservatorio Veterinario Italiano Cardiopatie, Azzano San Paolo, Italy; ^5^Bis Clinica Veterinaria Orobica Anicura, Bergamo, Italy; ^6^The Animal Medical Center, New York, NY, United States; ^7^Department of Biomedical Sciences and Cornell Veterinary Biobank, College of Veterinary Medicine, Cornell University, Ithaca, NY, United States; ^8^Department of Clinical Sciences, Faculty of Veterinary Medicine and Animal Science, Swedish University of Agricultural Sciences, Uppsala, Sweden; ^9^Wisdom Panel, Mars Petcare Science & Diagnostics, Leicestershire, United Kingdom; ^10^Veterinary Information Network and School of Veterinary Medicine and Epidemiology, University of California, Davis, Davis, CA, United States; ^11^Department of Clinical Sciences, Faculty of Veterinary Medicine, Utrecht University, Utrecht, Netherlands; ^12^Department of Veterinary Medicine and Surgery, College of Veterinary Medicine, University of Missouri, Columbia, MO, United States; ^13^Department of Veterinary Medicine and Animal Sciences, University of Milan, Lodi, Italy; ^14^Department of Animal Breeding and Genetics, Faculty of Veterinary Medicine and Animal Science, Swedish University of Agricultural Sciences, Uppsala, Sweden; ^15^Antagene, La Tour-de-Salvagny, France; ^16^Small Animal Department, Ghent University, Merelbeke, Belgium; ^17^Genefast srl, Forlì, Italy

**Keywords:** cardiac disease, feline genetics, variant classification, ACMG guidelines, genetic diversity

## Abstract

**Introduction:**

The correct labeling of a genetic variant as pathogenic is important as breeding decisions based on incorrect DNA tests can lead to the unwarranted exclusion of animals, potentially compromising the long-term health of a population. In human medicine, the American college of Medical Genetics (ACMG) guidelines provide a framework for variant classification. This study aims to apply these guidelines to six genetic variants associated with hypertrophic cardiomyopathy (HCM) in certain cat breeds and to propose a modified criterion for variant classification.

**Methods:**

Genetic samples were sourced from five cat breeds: Maine Coon, Sphynx, Ragdoll, Devon Rex, and British Short- and Longhair. Allele frequencies were determined, and in the subset with phenotypes available, odds ratios to determine the association with HCM were calculated. *In silico* evaluation followed with joint evidence and data from other publications assisting in the classification of each variant.

**Results:**

Two variants, MYBPC3:c.91G > C [A31P] and MYBPC3:c.2453C > T [R818W], were designated as pathogenic. One variant, MYH7:c.5647G > A [E1883K], was found likely pathogenic, while the remaining three were labeled as variants of unknown significance.

**Discussion:**

Routine genetic testing is advised solely for the MYBPC3:c.91G > C [A31P] in the Maine Coon and MYBPC3:c.2453C > T [R818W] in the Ragdoll breed. The human ACMG guidelines serve as a suitable foundational tool to ascertain which variants to include; however, refining them for application in veterinary medicine might be beneficial.

## 1 Introduction

Hypertrophic cardiomyopathy (HCM) is the most common heart disease in cats. Prevalence estimates vary between 3% (Pawpeds database, purebred cats, *n* = 19.866), 8% (purebred cats, *n* = 144), and even 15% (shelter cats, majority of domestic shorthairs and a small number of purebred cats, *n* = 780) (1–3). Based upon a 2012 estimate of feline pet ownership and extrapolating prevalence of 0.5% (= conservative estimate based on human prevalence estimates) and 8%, upwards of 470.000 to 7.5 million cats, respectively, could be affected by HCM (4, 5). Clinical signs can present as early as 3 months; although the median age of diagnosis has been reported at approximately 6.5 years, it may vary when evaluated in different populations (4, 6). HCM occurs in both sexes, in all breeds (but in some more frequently), and is characterized by primary concentric left ventricular hypertrophy with focal to diffuse myocardial thickening, myocardial fibrosis, and myocyte fiber disarray (7, 8). These features may lead to impaired diastolic filling, atrial dilation, congestive heart failure, thromboembolism, or sudden cardiac death (4, 9). Unless one or more of these events occur, affected cats may not express overt clinical signs, and many remain pre-clinical (10).

HCM in humans and cats shares several morphological and clinical similarities (11–14). HCM was first described as an autosomal dominant disorder in humans in 1960 (15). The first sarcomeric gene variant for HCM was identified in 1989 (16). Currently, more than 1700 gene variants have been associated with HCM in humans. The myosin-binding protein C3 (*MYBPC3*) gene and myosin heavy chain 7 (*MYH7*) gene contain most of the disease-associated variants (17). In domestic cats, six variants in four different genes (MYBPC3:c.91G > A [A31P], MYBPC3:c.220G > A [A74T], MYBPC3:c.2453C > T [R818W (XP_019667956.1); originally known a R820W], ALMS1:c.7384G > C [G2462R], TNNT2:c.95-108G > A, and MYH7:c.5647G > A [E1883]) have been reported to be associated with feline HCM (18–22).

While the association with HCM has been independently confirmed for some of the variants, this is not the case for all six variants, for example, the MYPBC3:c.220G > A [A74T] variant. This may in part be due to the lack of objective guidelines to evaluate variants’ pathogenicity. In human medicine, the American College of Medical Genetics (ACMG) guidelines are used for the interpretation and classification of sequence variants (23). Variants are classified into five different categories (pathogenic, likely pathogenic, unknown significance, likely benign, and benign) based on a comprehensive analysis that includes criteria such as functional studies, computational predictive models, co-segregation with the disease in families, and previously published research. Only when sufficient evidence is available to support the classification of the variant into one of the first two categories can a variant be used for clinical decision-making and genetic testing. The reason to only include variants that meet those criteria is obvious: the benefit of genetic tests is that they are quick and allow the early identification of cats predisposed to develop certain diseases (10). This early detection can be crucial for making informed breeding decisions and/or to decide at an early age which individuals should be retained as breeding stock (24). DNA tests based on variants that are not correctly associated with disease can lead to unnecessary exclusion of animals from breeding, can reduce confidence in DNA testing, and can create reluctance to use other reliable tests. Ultimately, this can reduce the effective population size and negatively impact the long-term health and diversity of animal populations (25). The objective of this study was to perform this evaluation of pathogenicity for the first time on all six variants reported to be associated with HCM using the existing human ACMG guidelines and to report a modified criterion that might help distinguish future variants in veterinary medicine.

## 2 Materials and methods

### 2.1 Study design

This study was established as a multicenter four-phase study and was based on (1) the prospective genotyping of samples of five breeds (British Short- and Longhair, Ragdoll, Sphynx, Maine Coon, and Devon Rex) for which the sample size requirements were met (see below), based on the samples stored at universities and repositories of Cornell University (USA), Ghent University (Belgium), Swedish University of Agricultural Sciences (Sweden), VetAgro Sup (France), Utrecht University (the Netherlands), and University of Milan (Italy) (= batch one) and (2) retrospective collection of data from two commercial laboratories (Antagene and Wisdom Panel) for the MYBPC3:c.91G > C [A31P] and MYBPC3:c.2453C > T [R818W] variants (= batch two). A subset of the samples provided by the Wisdom panel has been published in an earlier study with a different scope (26). The number of overlapping samples is depicted in Supplementary Table S1. For every breed in this study, these samples only represent a minority of what was available in the current study.

In the first phase, allele frequencies of all six published variants (the three in *MYBPC3* and one each in *TNNT2*, *MYH7*, and *ALMS1*) were determined. Inclusion criteria for samples used in this phase were (1) belonging to one of the five aforementioned breeds and (2) the necessary material had to be present to allow genotyping or genotypes had to be available. To be included in the second phase, an additional inclusion criterion had to be fulfilled: only samples for which a phenotype was available were used to calculate odds ratios (ORs) to evaluate the association between the variant and HCM. In this phase, three exclusion criteria were also used: one linked to the phenotype, one linked to the breed, and one linked to the genotype, as detailed in the statistical analysis section. In the third phase, all variants were assessed with *in silico* tools to predict their pathogenicity. In the final phase, the aforementioned evidence, together with additional data from other publications, was used to classify each variant using the ACMG guidelines.

### 2.2 Power analysis

A power analysis was conducted with the R package epiR to determine the minimal sample size necessary to detect a variant with a certain odds ratio (27). The odds ratio used for these calculations was based on the allelic odds ratio derived from the paper in which the variant was described for the first time. The lowest and highest disease prevalence reported in literature were used, leading to a HCM prevalence of 3% (= worst case scenario in terms of sample size) or 15% (= best case scenario in terms of sample size) in the group without the disease-causing allele, respectively (1, 3). The prevalence that resembled the prevalence in our sample most closely was retained. Furthermore, a balanced number of affected and healthy cats and a desired power of 0.80 were assumed. As the odds ratio differed for each variant, the required total sample sizes ranged between 4 and 32. An overview for each variant is presented in Supplementary Table S2.

### 2.3 Ethics

For batch one, all included cats had informed owner consent. For batch two, the owners provided written consent for data use in research upon submission of samples for commercial genetic testing. Samples were non-invasive buccal swabs or whole blood on EDTA. In some cats, blood was obtained as part of routine clinical procedures for diagnostic purposes, at the request and with the consent of the owner. As these samples were from client-owned cats for which no harmful invasive procedures were performed, there was no animal experimentation according to the legal definition in Europe (Subject 5f of Article1, Chapter I of the Directive 2010/63/UE of the European Parliament and of the Council). A subset of the blood samples was collected as part of a biobanking initiative, approved by the Ethics Committee of the Faculties of Veterinary Medicine and Bioscience Engineering at Ghent University (EC2017-86). Samples from Sweden were collected in agreement with the ethical committee of the Swedish Board of Agriculture [No. C2/12 (2012-02-24),C12/15 (2015-02-27), and Dnr 5.8.18–04682/2020]. Samples sourced from the Cornell Veterinary Biobank were collected under Institutional Animal Care and Use Committee (IACUC) approved animal protocol #2005–0151.

### 2.4 Genotyping

A detailed overview of the primers, product lengths and type of test, PCR temperatures, and other parameters used for genotyping of the samples of batch one can be found in Supplementary Table S3. For ALMS1:c.7384G > C, the recently published primers were used to avoid allelic drop-out (28). A subset of the samples of batch two (*n* = 10.851) were genotyped using a commercial custom genotyping microarray [i.e., MyCatDNA and the Optimal Selection Feline tests (26)].

### 2.5 Phenotyping

HCM phenotyping was performed by echocardiography according to the American College of Veterinary Internal Medicine (ACVIM) consensus guidelines for classification, diagnosis, and management of cardiomyopathy in cats (29). Echocardiography was performed by board-certified veterinary cardiologists, residents under direct supervision of a cardiologist or experienced cardiology clinicians. An HCM phenotype was defined as the presence of diffuse or regional increased LV wall thickness with a non-dilated LV chamber. A combination of two-dimensional (2D)-guided M-mode (right parasternal short axis view) and 2D echocardiography (right parasternal long axis view and right parasternal short axis view) was used to measure LV wall thickness. For M-mode, and for 2D measurement of the LV free wall, end-diastolic wall thickness was measured using a leading edge-to-leading edge technique, whereas a leading edge-to-trailing edge technique was applied for 2D measurement of interventricular septum thickness. At least three cardiac cycles were measured and averaged. Left atrial size was measured via 2D echocardiography in the right parasternal heart base view (left atrium-to-aorta ratio) and right parasternal long axis view (left atrial long axis diameter). Additional features of HCM were also assessed, including papillary muscle hypertrophy, end-systolic chamber obliteration, septal or apical hypertrophy, mitral valve systolic anterior motion, left ventricular outflow tract obstruction, and spontaneous echocontrast. Diastolic function was investigated using pulsed-wave and tissue Doppler imaging. In the realm of feline cardiology, there is no universally accepted standard for determining the normal thickness of the left ventricular wall during diastole. It is overly simplistic to assume that a single numerical value can reliably distinguish between a healthy left ventricular wall and one affected by hypertrophy across all cats. The thickness of the left ventricular wall increases nonlinearly with body weight and is influenced by physiological factors such as hydration level and heart rate. Conditions like hyperthyroidism and systemic hypertension, which are common in older cats, can further impact left ventricular wall thickness, and the presence of these conditions were examined in cats, if suspected, before inclusion.

Nevertheless, for most cats that are not hyperthyroid or hypertensive, of average size and with a normal body condition (weighing between 3.5–5 kg), a left ventricular diastolic wall thickness of 5 mm or less is typically considered within the normal range, while a thickness of 6 mm or more suggests concentric hypertrophy. Cats with a left ventricular wall thickness falling between 5 and 6 mm were termed equivocal and were excluded from analysis. Cats outside this weight range were managed on a case to case basis with the aid of previously published 95% prediction intervals (1).

### 2.6 Statistical analysis

All analyses were performed in R version 4.1.1. and Excel version 16.54. Allele frequencies were calculated for each breed separately. In the second phase of our study, where ORs and their 95% confidence intervals were computed, only cats for which phenotypical data were available were included. Cats with ambiguous hypertrophic cardiomyopathy (equivocal cats), cats diagnosed with restrictive cardiomyopathy, and healthy cats under 6 years of age were excluded (phenotypical exclusion criteria). This age cutoff for the control group was based on the observation that median or mean age of diagnosis lies approximately at 6 years (4, 30). By setting this as the minimal age, the goal was to obtain a control group in which the overall age would actually be higher than the cutoff as an increased age reduces the probability of misclassifying cats further. The association of each individual variant with HCM was determined (1) in the breed in which the variant was originally described (breed exclusion criterion) and (2) if a variant was not significant, this analysis was repeated in all breeds in which the variant segregated. If several variants were originally described in a breed (i.e., the three variants reported in the Maine Coon), only homozygous wild-type individuals for those variants except the one that was assessed were included to avoid the potential influence of these other variants. For the analysis across breeds, cats had to be homozygous wild type for all variants other than the variant that was evaluated. The exclusion based on genotype thus represents the genotypic exclusion criterion. Both allelic ORs (i.e., the ratio between the odds of having the phenotype and a specific allele and the odds of the same phenotype without having the allele) and genotypic ORs (based on two inheritance patterns, i.e., autosomal dominant and autosomal recessive) were calculated for every variant, using unconditional maximum likelihood estimation and normal approximation [Wald] confidence intervals. The Haldane-Anscombe correction was applied when necessary (31).

### 2.7 *In silico* analysis

For each variant, *in silico* computational prediction methods were employed using three online tools. For every variant, except TNNT2:c.95-108G > A, the following missense prediction tools were used: Polyphen-2^1^, Sift^2^ (default settings), and Mutpred2^3^ (*p*-value threshold: 0.05). The MYBPC3 [uniprot: M3VYP3_FELCA, (XM_019812396.1 RefSeq)] protein sequence was used for the three MYBPC3 variants (MYBPC3:p.A31P, MYBPC3:p.A74T, MYBPC3:p.R818W). The MYH7 (XM_006932746.4 RefSeq) protein sequence was used for the MYH7:p.E1883K variant. The ALMS1 (A0A5F5XP77_FELCA) protein sequence was used for the ALSM1:c.7384G > C [G2462R] variant.

For the TNNT2:c.95-108G > A variant, the following tools were used to predict the effect on mRNA for splicing of the TNNT2 gene: ASSP^4^ (default parameters), GENSCAN^5^ (organism: vertebrate), Netgene2^6^ (organism: Human). The TNNT2 genomic sequence (ENSFCAG0000004613) was used both with and without the variant as input for each tool. Wild type and variant sequence predicted splice sites were compared.

### 2.8 The American College of Medical Genetics classification

The ACMG has developed a systematic approach for classifying genetic variants, based on a set of criteria. Each criterion has a specific weight, and the set of fulfilled criteria determines the final classification, which ranges from pathogenic to benign. A concise overview of the criteria that were relevant is available in Supplementary Table S4. The classification of the variants into five categories (pathogenic, likely pathogenic, variant of unknown significance, likely benign, and benign) was done in two stages. In the first stage, the ACMG guidelines were used except for the criteria related to allele frequency, in agreement with what has been done in earlier studies (21, 32, 33). In the second stage, the additional effect of using these allelic frequency criteria for classification was evaluated. In this second stage, an alternative cutoff, i.e., the alternative allele frequency threshold (Tv), was also calculated and used (Supplementary Table S5) (34). This alternative cutoff was used with the same weight as in the ACMG guidelines (i.e., a stand-alone benign (BA), a strong benign (BS), or a moderate pathogenic (PM) criterion). To allow distinction with the original criteria, they are denoted as “BA1*,” “BS1*,” and “PM2*” instead of the original “BA1,” “BS1,” and “PM2” allelic frequency cutoff related criteria, respectively.

## 3 Results

### 3.1 Allele frequencies

For the calculation of allelic frequencies (Table 1), all available samples regardless of phenotype were used. The calculation of allelic frequencies was based on the combination of samples from the universities/repositories (=batch one) and data from commercial laboratories (=batch two), regardless of whether a phenotype was available. From batch one, the number of samples per breed was 45 British Short- and Longhair, 20 Ragdoll, 107 Sphynx, 97 Maine Coon, and 33 Devon Rex cats. These were complemented by screening results from two commercial laboratories that have already tested for MYBPC3:c.91G > C [A31P] and MYBPC3:c.2453C > T [R818W] for several years, increasing the sample sizes for those two variants up to nearly a thousand to several thousand samples, depending on the breed. An overview of the sample sizes is provided in Table 1. Furthermore, the evolution over time for MYBPC3:c.91G > C [A31P] and MYBPC3:c.2453C > T [R818W] was available for a subset of the samples from one commercial laboratory and is depicted in Supplementary Table S6.

**Table 1:** Overview of the allele frequencies of the six variants reported to be associated with hypertrophic cardiomyopathy in cats.

Variant	Total	Wt/Wt	Wt/Vt	Vt/Vt	*q* (%)
*British Short- and Longhair*					
MYBPC3:c.91G > C [A31P]	878	878	0	0	0
MYBPC3:c.220G > A [A74T]	45	14	20	11	46.67
MYBPC3:c.2453C > T [R818W]	878	878	0	0	0
TNNT2:c.95-108G > A	45	42	3	0	3.33
ALMS1:c.7384G > C [G2462R]	45	44	1	0	1.11
MYH7:c.5647G > A [E1883K]	44*	44	0	0	0
*Ragdoll*					
MYBPC3:c.91G > C [A31P]	2,979	2,978	1	0	0.02
MYBPC3:c.220G > A [A74T]	20	13	6	1	20
MYBPC3:c.2453C > T [R818W]	4,754	4,533	212	9	2.42
TNNT2:c.95-108G > A	20	14	6	0	15
ALMS1:c.7384G > C [G2462R]	20	19	1	0	2.5
MYH7:c.5647G > A [E1883K]	20	20	0	0	0
*Sphynx*					
MYBPC3:c.91G > C [A31P]	1,159	1,159	0	0	0
MYBPC3:c.220G > A [A74T]	106*	9	72	25	57.55
MYBPC3:c.2453C > T [R818W]	1,159	1,159	0	0	0
TNNT2:c.95-108G > A	107	99	8	0	3.74
ALMS1:c.7384G > C [G2462R]	107	24	55	28	51.87
MYH7:c.5647G > A [E1883K]	104*	104	0	0	0
*Maine Coon*					
MYBPC3:c.91G > C [A31P]	14025*	12,508	1,444	75	5.68
MYBPC3:c.220G > A [A74T]	95*	59	29	7	22.63
MYBPC3:c.2453C > T [R818W]	5,181	5,181	0	0	0
TNNT2:c.95-108G > A	97	51	39	7	27.32
ALMS1:c.7384G > C [G2462R]	97	96	0	1	1.03
MYH7:c.5647G > A [E1883K]	77*	77	0	0	0
*Devon Rex*					
MYBPC3:c.91G > C [A31P]	956	956	0	0	0
MYBPC3:c.220G > A [A74T]	32*	7	22	3	43.75
MYBPC3:c.2453C > T [R818W]	956	956	0	0	0
TNNT2:c.95-108G > A	33	30	3	0	4.69
ALMS1:c.7384G > C [G2462R]	33	15	14	4	32.81
MYH7:c.5647G > A [E1883K]	33	33	0	0	0

Per breed, the total number of cats tested (Total), the number of homozygous wild type (Wt/Wt), heterozygous (Wt/Vt), and homozygous variant (Vt/Vt) individuals, as well as the corresponding variant allele frequency (q) are reported, respectively. *Genotype information was missing for this variant in some samples. In more detail, one British Short- and Longhair had no information on the MYH7:c.5647G > A variant. One Sphynx had no genotype for the MYBPC3:c.220G > A variant. There were four Maine Coons with missing data, two for the MYBPC3:c.91G > C and two for the MYBPC3:c.220G > A variant, respectively. Thirteen Maine Coons and three Sphynx had missing genotypes for the MYH7:c.5647G > A variant. Finally, one Devon Rex did not have genotype data for the MYBPC3:c.220G > A variant.

Overall, one variant (MYH7:c.5647G > A [E1883K]) was not observed in any cat, one variant (MYBPC3:c.2453C > T [R818W]) was found only in the Ragdoll cats, and the MYBPC3:c.91G > C [A31P] variant segregated in the Maine Coon and one Ragdoll. The remaining three variants segregated in all five breeds.

### 3.2 Association with HCM

Twenty-nine British Short- and Longhair (cases = 16, controls = 13), 16 Ragdoll (cases = 9, controls = 7), 84 Sphynx (cases = 53, controls = 31), 92 Maine Coon (cases = 47, controls = 45), and 25 Devon Rex (cases = 15, controls = 10) were retained after the application of the phenotypical inclusion and exclusion criteria. Subsequently, for the first analysis focusing on the individual breeds in which the variants were initially identified (=Maine Coon, Ragdoll, and Sphynx; the breed exclusion criterion), we restricted our sample to cats that were homozygous for the ‘wild-type’ variant of all other gene variants under investigation in that particular breed. This (genotype) exclusion criterion was put in place to prevent any confounding influence from other variants. After eliminating all non-homozygous wild-type cats for the MYBPC3:c.220G > A [A74T] and TNNT2:c.95-108G > A variants, the total number of Maine Coons available for the OR calculation of MYBPC3:c.91G > C [A31P] was 37. By employing the same methodology, 27 cats and 28 cats were retained for the MYBPC3:c.220G > A [A74T] and TNNT2:c.95-108G > A variant, respectively. The sample sizes for each variant, as well as the median age and the range for each of the three breeds in which the variants were originally found, are reported in Table 2. Overall, the median age for the cases was 4 years (range: 0.25–16.50), while the median age for the controls was 8 years (range: 6.00–23.00).

**Table 2:** Overview of the allelic and genotypic odds ratios (ORs) of the six variants reported to be associated with HCM in cats.

Variant	*n*	Case			Control			IP	OR	CI
		Wt/Wt	Wt/Vt	Vt/Vt	Wt/Wt	Wt/Vt	Vt/Vt			
*Maine Coon*										
Median age		5 [0.83–16.5]			10 [6.92–23]					
MYBPC3:c.91G > C [A31P]	37	8	3	9	10	6	1	**A**	**3.59**	**[1.31–9.83]**
								**R**	**13.09**	**[1.44–118.62]**
								D	2.14	[0.57–7.99]
MYBPC3:c.220G > A [A74T]	27	8	5	1	10	3	0	A	2.56	[0.58–11.18]
								R	3	[0.11–80.40]
								D	2.5	[0.47–13.27]
TNNT2:c.95-108G > A	28	8	4	2	10	3	1	A	1.84	[0.52–6.54]
								R	2.17	[0.17–27.08]
								D	1.88	[0.39–9.01]
*Ragdoll*										
Median age		2 [1.08–10.3]			8 [7.67–10.8]					
MYBPC3:c.2453C > T [R818W]	16	4	0	5	7	0	0	**A**	**35.82**	**[1.85–692]**
								R	18.33	[0.81–416.06]
								D	18.33	[0.81–416.06]
*Sphynx*										
Median age		3 [0.25–12]			8 [6.08–12.5]					
ALMS1:c.7384G > C [G2462R]	84	12	25	16	6	17	8	A	1.02	[0.55–2]
								R	1.24	[0.46–3.36]
								D	0.82	[0.27–2.46]

The genotypic OR that corresponds with the mode of inheritance proposed in the original paper is underlined. Significant ORs are bold. The median age in years (and range) of cases and controls is reported for each breed. n, total number of cats; Wt/Wt, homozygous wild type; Wt/Vt, heterozygous; Vt/Vt, homozygous variant; IP, inheritance pattern; OR, odds ratio; CI, 95% confidence interval; A, allelic OR; R, autosomal recessive genotypic OR; D, autosomal dominant genotypic OR.

A significant allelic OR was found for the MYBPC3:c.91G > C [A31P] variant and the MYBPC3:c.2453C > T [R818W] variant. A significant genotypic OR was also found for the MYBPC3:c.91G > C [A31P] (autosomal recessive mode of inheritance). None of the other variants were significantly associated with HCM. ORs could not be calculated for the MYH7:c.5647G > A [E1883K] variant due to its absence in the dataset. For the three variants that were not significant (MYBPC3:c.220G > A [A74T], TNNT2:c.95-108G > A and ALMS1:c.7384G > C [G2462R]), the analysis was repeated across all breeds in which the variant was found. However, none of the point estimates increased (Supplementary Table S7).

### 3.3 Prediction of harmful effects

Two sets of *in silico* computational prediction methods were used to investigate the impact of genetic variants on protein function and mRNA splicing, respectively. The algorithms were only consistent for MYBPC3:c.2453C > T [R818W] (predicted pathogenic), ALMS1:c.7384G > C [G2462R] (predicted pathogenic), and MYH7:c.5647G > A [E1883K] (predicted pathogenic). For the other three variants, the algorithms gave inconsistent results (Table 3).

**Table 3:** Overview of the outcomes of six hypertrophic cardiomyopathy-associated variants analyzed with *in silico* prediction tools.

Tool	Result	Conclusion
MYBPC3:c.91G > C [A31P]		
Polyphen-2	Probably damaging	Inconsistent
Sift	Predicted tolerated	
Mutpred2*	0.698	
MYBPC3:c.220G > A [A74T]		
Polyphen-2	Probably damaging	Inconsistent
Sift	Predicted tolerated	
Mutpred2*	0.114	
MYBPC3:c.2453C > T [R818W]		
Polyphen-2	Probably damaging	Consistent (pathogenic)
Sift	Predicted not tolerated	
Mutpred2*	0.631	
TNNT2:c.95-108G > A		
ASSP	*De novo* acceptor site	Inconsistent
GENSCAN	No extra exons created	
Netgene2	No effect	
ALMS1:c.7384G > C [G2462R]		
Polyphen-2	Probably damaging	Consistent (pathogenic)
Sift	Predicted not tolerated	
Mutpred2*	0.586	
MYH7:c.5647G > A [E1883K]		
Polyphen-2	Probably damaging	Consistent (pathogenic)
Sift	Predicted not tolerated	
Mutpred2*	0.782	

*A mutpred2 score exceeding 0.5 indicates a pathogenic mutation.

### 3.4 ACMG variant classification

The combination of the ORs, the results of the predictive algorithms, and publications regarding functional studies and evidence across species was used to classify the variants with the ACMG guidelines (Tables 4, 5). In addition, the allelic frequencies found here were compared with the calculated Tvs (called “PM2*,” “BA1*,” and “BS1*,” respectively) and the ACMG criteria related to allelic frequency cutoffs (called “PM2,” “BA1,” and “BS1,” respectively). As the assignment to one of the five categories (from pathogenic to benign) depends on the combined evidence of various criteria and as individual criteria have different weights, one additional criterion can sometimes be sufficient to alter the assigned category. As such, the impact of these comparisons on the final classification is illustrated in Table 6.

**Table 4:** Summary of the relevant variant classification criteria and the final category for each of the variants located in the *MYBPC3* gene.

	Criterion	Remark	Result
MYBPC3:c.91G > C [A31P]			
**PS4**	Significant OR > 5.0	OR (AR): 13.09 [1.44–118.62]	F
**PP3**	Multiple lines of computational evidence	1/3 predicted no harmful effects	N
**PS3**	Well-established functional studies	Reduced MYBPC3 in affected cats confirmed by 3 independent techniques in original article. Sarcomeric protein organization altered in affected cats (18).	F
**PP2**	Missense variant in a gene with common mechanism of disease	Variants in human *MYBPC3* are the most common cause of HCM (35).	F
Classification: pathogenic			
MYBPC3:c.220G > A [A74]			
**PS4**	Significant OR > 5.0	No significant allelic or genotype OR	N
**PP3**	Multiple lines of computational evidence	2/3 predicted no harmful effects	N
**PS3**	Well-established functional studies	No confirmation on mRNA or protein level. The variant does not seem to alter any structural feature of the MYBPC3 protein (36).	N
**PP2**	Missense variant in a gene with common mechanism of disease	Variants in human *MYBPC3* are the most common cause of HCM (35).	F
Classification: unknown significance			
MYBPC3:c.2453C > T [R818W]			
**PS4**	Significant OR > 5.0	Allelic OR: 35.82 [1.85–692]	F
**PP3**	Multiple lines of computational evidence	3/3 predicted harmful effects	F
**PS1**	Same amino acid change as a previously established pathogenic variant regardless of nucleotide change	The R818W variant was found to cause HCM in homozygous human carriers, with mild or null clinical expression in heterozygous carriers (37)	F
**PS3**	Well-established functional studies	/	N
**PM5**	Novel missense change at an amino acid residue where a different missense change is determined to be pathogenic has been seen before	Elderly human patients with an R818Q MYBPC3 missense mutation show burnt-out phase of HCM (37).	F
**PP2**	Missense variant in a gene with common mechanism of disease	Variants in human *MYBPC3* are the most common cause of HCM (35).	F
Classification: pathogenic			

HCM, hypertrophic cardiomyopathy; OR, odds ratio; AR, autosomal recessive; F, fulfilled; N, not fulfilled.

**Table 5:** Summary of relevant variant classification criteria for the variants located in the *TNNT2*, *ALMS1*, and *MYH7* genes.

	Criterion	Remark	Result
TNNT2:c.95-108G > A			
**PS4**	Significant OR > 5.0	No significant allelic or genotype OR	N
**PP3**	Multiple lines of computational evidence	2/3 predicted no harmful effects	N
**PVS1**	Null variant in a gene where LOF is a known mechanism	LOF possible in TNNT2, LOF not confirmed on mRNA or protein level (33).	N
Classification: unknown significance			
ALMS1:c.7384G > C [G2462R]			
**PS4**	Significant OR > 5.0	No significant allelic or genotype OR	N
**PP3**	Multiple lines of computational evidence	3/3 predicted harmful effects	F
**PS3**	Well-established functional studies	The function of the protein is not well understood although it is believed to be associated with energy metabolism and homeostasis, intracellular trafficking, cell signaling pathways, cell differentiation and cell cycle control. Extra functional studies needed (20).	N
Classification: unknown significance			
MYH7:c.5647G > A [E1883K]			
**PP3**	Multiple lines of computational evidence	3/3 predicted harmful effects	F
**PS3**	Well-established functional studies	A 29 residue region (residues 1874–1902) is necessary for ordered paracrystal formation and is sufficient to confer assembly properties to an assembly incompetent rod fragment (38). Furthermore, E1886K has a decreased ability to assemble and paracrystals formed from this mutant protein are much more readily degraded than those formed from WT protein (39). Expression of E1883K mutant protein in a *Drosophila* animal model causes ultrastructural thick filament misalignment, disrupted sarcomere structure in pupae and depress flight and jump ability (40). This does not seem to affect motility in *C. elegans* worm (41).	F
**PM1**	Located in mutational hot spot and/or critical and well-established functional domain without benign variation	A significant cluster was observed between residues 181 and 937 in HCM cases (17).	N
**PP2**	Missense variant in a gene with common mechanism of disease	Variants in human *MYH7* are a common cause of HCM (35, 42).	F
Classification: likely pathogenic			

HCM, hypertrophic cardiomyopathy; LOF, loss of function; OR, odds ratio; F, fulfilled; N, not fulfilled.

**Table 6:** Summary of the classification of the variants reported to be associated with hypertrophic cardiomyopathy with and without using the allelic frequency (AF) criteria based on the American College of Medical Genetics and Genomics (ACMG) guidelines or the alternative allelic frequency threshold (Tv), respectively.

	Criterion	Remark
MYBPC3:c.91G > C [A31P]		
Initial classification: pathogenic (PS4 + PS3 + PP2)		
**ACMG: BA1**	AF > 5%	AF (Maine Coon) = 5.6%
Classification with BA1: benign		
Tv: PM2*	Alternative Tv: 17%	AF (Maine Coon) = 5.6%
Classification with PM2*: pathogenic		
MYBPC3:c.220G > A [A74T]		
Initial classification: unknown significance (PP2)		
**ACMG: BA1**	AF > 5%	AF (Maine Coon): 22.63%. Variant allele is present in every breed studied in this study.
Classification with BA1: benign		
Tv: PM2*	Alternative Tv: 40%	AF (Maine Coon) = 22.63%
Classification with PM2*: unknown significance		
MYBPC3:c.2453C > T [R818W]		
Initial classification: pathogenic (PS4 + PP3 + PS1 + PM5 + PP2)		
**ACMG: PM2**	AF < 5%	Only present in Ragdolls with an AF = 2.4%
Classification with PM2: pathogenic		
Tv: PM2*	Alternative Tv: 11%	AF (Ragdoll) = 2.4%
Classification with PM2*: pathogenic		
TNNT2:c.95-108G > A		
Initial classification: unknown significance		
**ACMG: BA1**	AF > 5%	AF (Maine Coon) = 27.32%. Variant allele is present in every breed studied in this study.
Classification with BA1: benign		
Tv: BS1*	Alternative Tv: 27%	AF (Maine Coon) = 27.32%
Classification with BS1*: unknown significance		
ALMS1:c.7384G > C [G2462R]		
Initial classification: unknown significance (PP3)		
**ACMG: BA1**	AF > 5%	AF (Sphynx) = 51.87%. Variant allele is present in every breed studied in this study.
Classification with BA1: benign		
Tv: BS1*	Alternative Tv: 15%	AF (Sphynx) = 51.87%
Classification with PM2*: unknown significance		
MYH7:c.5647G > A [E1883K]		
Initial classification: likely pathogenic (PP3 + PS3 + PP3)		
**ACMG: PM2**	AF < 5%	Absent from dataset
Classification with BA1: pathogenic		
Tv: PM2*	Alternative Tv: 2%	Absent from dataset
Classification with PM2*: pathogenic		

The criteria that were fulfilled and led to the initial classification are mentioned in between brackets. F, fulfilled; N, not fulfilled; *Tv calculations can be found in Supplementary Table S3.

For MYBPC3:c.91G > C [A31P], the combination of three pathogenic criteria results in a final classification as pathogenic (Table 4). While the allele frequency of 5.6% would result in an immediate classification as benign based on the allelic frequency criteria from the ACMG (i.e., “BA1”), the frequency is lower than the alternative Tv. As such, the alternative Tv would have led to additional support (“PM2*”) for the classification of pathogenic, while the ACMG criteria conflict with this evaluation (Table 6).

For MYBPC3:c.220G > A [A74T], there was only one pathogenic criterion fulfilled (Table 4). As such, the variant is classified as a variant of unknown significance. While the allele frequency of 22.63% would result in an immediate classification as benign based on the allelic frequency criteria from the ACMG (i.e., “BA1”), the frequency is lower than the alternative Tv (leading to additional “PM2*” support). This would, however, not be sufficient evidence to change the classification to likely pathogenic (Table 6).

For MYBPC3:c.2453C > T [R818W], five pathogenic criteria were fulfilled, resulting in classification as pathogenic (Table 4). With an allele frequency of 2.4%, both the allelic frequency criteria (i.e., “PM2”) and the Tv (i.e., “PM2*”) support the classification as pathogenic (Table 6).

For TNNT2:c.95-108G > A, there were no pathogenic or benign criteria fulfilled, resulting in a classification as a variant of unknown significance (Table 5). With an allele frequency > 27% in the Maine Coon, the allelic frequency criteria of the ACMG alone (i.e., “BA1”) would immediately result in a classification as benign, while the Tv would add additional evidence for benign (i.e., “BS1*”) but this would be insufficient to change the classification to likely benign.

For ALMS1:c.7384G > C [G2462R], only one pathogenic criterion was fulfilled, resulting in a classification as a variant of unknown significance (Table 5). With an allele frequency of close to 52%, the allelic frequency criteria of the ACMG alone (i.e., “BA1”) would immediately result in a classification as benign, while the Tv would add additional evidence for benign (i.e., “BS1*”) but this would be insufficient to change the classification to likely benign.

For MYH7:c.5647G > A [E1883K], three pathogenic criteria were fulfilled, resulting in classification as likely pathogenic (Table 5). With an allele frequency of 0%, both the allelic frequency criteria of the ACMG (“PM2”) and the Tv (“PM2*”) would support the classification as likely pathogenic.

## 4 Discussion

As a general trend, tests based on genetic variants are often quickly commercially available after their discovery and used for breeding decisions. Compared to phenotypic testing, genetic tests have several advantages, but only when the association these tests are based on is valid. As several variants for HCM were only recently discovered, and as there is a history of spurious associations [i.e., for MYBPC3:c.220G > A [A74T] and TNNT2:c.95-108G > A, the initial associations have not been replicated in subsequent studies (33, 36)], our goal was to classify all six currently published variants based on the ACMG guidelines.

To do this, this study focused on cats of five breeds: the Maine Coon, Ragdoll, Sphynx, British Short- and Longhair, and the Devon Rex. The reasons were that (1) they represent the breed in which a variant was originally discovered (the first three) and/or, (2) because a breed predisposition for HCM has been reported (the first three and the British Short- and Longhair) and/or, (3) because it has been crossbred with the Sphynx (the Devon Rex, British Short- and Longhair). While there are other breeds predisposed to develop HCM, it was decided upfront to only include those breeds in which the sample size requirements were met to allow the association analysis to be performed with sufficient power. An evaluation of the various contributors of the samples shows that the origins of the samples are diverse: the largest number of the samples used in the allelic frequency part of the study originates from laboratories that commercially offer DNA testing, while others originate from a clinical work-up, which can involve screening, diagnostics, and so on. It is difficult to assess whether this might have biased the estimates: samples obtained in a clinical setting can lead to a higher prevalence of disease (43). However, also samples obtained from a genetic testing facility can be biased compared to samples obtained randomly from the population (44). From a practical point of view, it is difficult to truly randomly collect samples. Obtaining sample sizes similar to this study would have required an enormous effort to reach out to groups of cats throughout the world. Furthermore, even then, it cannot be excluded that owners interested in HCM are predominantly submitting samples. Also ethically, the approach is not ideal as it would likely have led to additional invasive sampling, at least if sampling would not be restricted to swabs. Overall, the current method of sampling seemed to be the most convenient and allowed us to collect a sample size of well over 10.000 cats. Furthermore, as discussed further, the allele frequency estimates are in agreement with other studies, which indicates that if there was a bias, it did not result in unexpected deviations.

The first three phases of this study dealt with determining allelic frequencies, ORs, and *in silico* predictions, respectively. As these results were used to subsequently classify the variants, it is important to assess whether our findings are in line with previous reports, if available.

In terms of allelic frequency, the MYBPC3:c.91G > C [A31P] variant was found in the Maine Coon and one Ragdoll. The presence of the variant in a Ragdoll was a new finding compared to what has been reported in the literature. Considering that the sample size was substantial in the current study (and to our knowledge, the highest reported yet) and that it was only observed in a heterozygous state in one Ragdoll, this seems to be an extremely rare case. Recently, this variant was also found in unique cases in additional breeds: one Pixie-Bob, one Siberian, one Scottish Fold, and one Munchkin (26, 45). These rare occurrences may not necessarily indicate a natural prevalence in these breeds. Instead, they could also result from (1) outcrossing with Maine Coon cats, as has been suggested in previous genetic studies (38), or (2) faulty description of the breed. A third option, a genotyping error, was deemed very unlikely as the raw data of the sample were manually checked and looked entirely normal and the genotype result for that variant is perfectly consistent across various replicate assays. Overall, these findings are notable, but the interpretation of their significance should be made cautiously. The allelic frequency in the Maine Coon was 6% in the present study. In the literature, allelic frequencies in the Maine Coon were slightly higher, with values ranging between 11 and 41%. The highest values were, however, found in the oldest studies, while the allelic frequency was lowest in the most recently published study (26, 36, 46–50). We hypothesized that this might have been the result of selection against this variant as it was discovered nearly two decades ago (18). The data from one of the commercial laboratories that contributed to this study were consistent with this hypothesis: their data showed that allelic frequencies decreased substantially from 2008 to 2021 from 19.54 to 0.95% and from 15.63 to 0% for the MYBPC3:c.91G > C [A31P] and the MYBPC3:c.2453C > T [R818W] variant, respectively. This aligns with the temporal trends observed in earlier research and is slightly lower in 2021 than our current estimate (Supplementary Table S6) (36, 46–50). Overall, in agreement with the literature, this is a variant that mainly occurs in the Maine Coon breed, accidentally in other breeds and selection may have reduced its frequency. The MYBPC3:c.220G > A [A74T] variant was found in all five breeds, with allelic frequencies ranging between 22 and 58%. In the literature, it has also been observed in Norwegian Forest, Persian, domestic shorthair, Siberian, and Bengal cats, with allelic frequencies ranging between 17 and 100% (36, 48). Overall, this variant seems widespread and occurs at high frequencies. The MYBPC3:c.2453C > T [R818W] variant was only found in Ragdoll cats in the current study, with an allelic frequency of 2%. While the allelic frequency in the literature also tended to be higher, ranging between 14 and 23% (36, 46), a more recent study showed an allele frequency of 3% in Ragdolls (26). Recently, this variant was also found in the American Bobtail (with an allele frequency of 11.4% in the longhaired variant), one Highlander, One Munchkin, and two RagaMuffins, all breeds in which crosses to Ragdolls possibly have been part of breed development (26). As allelic frequencies over nearly 15 years of selection have declined (Supplementary Table S6), a trend similar to that for MYBPC3:c.91G > C [A31P] is observed, which might be a consequence of breeder selection (33, 36, 46–48). The TNNT2:c.95-108G > A was found in all five breeds, with allelic frequencies ranging between 2 and 27%. In addition to the breeds studied here, the variant has also been found previously in Persian, Siamese, Tennessee Rex, and Thai breeds, as well as domestic shorthair cats and allelic frequencies were very similar to this study, ranging between 1 and 25% (33). Hence, this is a widespread variant, occurring at high allelic frequencies. MYH7:c.5647G > A [E1883K] was only found in one domestic shorthair cat in the initial report, and the variant has not been found here, nor in a different study of 103 HCM-affected cats (47). Here, no domestic shorthairs were included, so as a result, we can only state that the variant does not seem to segregate in the breeds included in this study. Overall, this seems to be an example of a rare variant. The ALMS1:c.7384G > C [G2462R] variant was observed here in all five breeds studied, at allelic frequencies ranging between 1 and 52%. In the original study, this variant was also reported to segregate in two non-Sphynx cats, which may have belonged to any of 14 breeds included in that study (20). Four of these 14 breeds are also present in this study. Recently, the variant has been reported in the Sphynx at allelic frequencies >50% and also reported in the Scottish Fold, the Exotic Shorthair, and the Minuet breeds, at allelic frequencies approximately 8–9% (28, 45). Hence, this is a widespread variant occurring at high allelic frequencies.

Overall, in terms of allelic frequencies and breeds in which the variant is reported, our data seem to be consistent with the literature and variant characteristics seem to range from rare in terms of number of breeds (MYBPC3:c.2453C > T [R818W], MYBPC3:c.91G > C [A31P]), allelic frequency, or both (MYH7:c.5647G > A [E1883K]) to extremely widespread and prevalent (MYBPC3:c.220G > A [A74T], TNNT2:c.95-108G > A and ALMS1:c.7384G > C [G2462R]).

In our study, a critical aspect was the evaluation of ORs. This is a fundamental tool for the quantification of the association between variants and phenotypes. Nevertheless, various challenges presented, stemming from methodological considerations, sample size constraints, and inconsistencies in the literature. First of all, various calculation methods exist for ORs: one can start from the alleles themselves, or start with the genotypes and group them according to a prespecified inheritance model (51). Instead of choosing one method, we opted for performing several. The reason is that inheritance modes were not always clear in the respective original study or were sometimes disputed in follow-up studies. The downside of this strategy is the multiple testing issue, i.e., the type I error rate or number of false positives increases. As correcting for this negatively affects the power, we chose not to perform a correction because one potential criticism might be that by deliberately increasing the number of tests, we missed out on correct associations. Second, due to the high allelic frequency of several variants (especially MYBPC3:c.220G > A [A74T], ALMS1:c.7384G > C [G2462R], and TNNT2:c.95-108G > A), the sample size quickly decreased when only individuals who were wild type for the other important variants in that breed were retained. Our initial sample size was sufficiently high, so this was not a pressing issue in the current study. However, as the number of potentially associated variants increases in future studies—especially if they are as common as the ones mentioned—securing an adequate sample size will likely become more challenging. A final remark is that a comparison with literature was difficult, as ORs were not always reported and if they were reported, it was not always specified how they were calculated. Nevertheless, for several variants, we were able to compare our results with previous reports in the literature.

For MYBPC3:c.91G > C [A31P], the initial allelic OR in one study was 62.14 (18). The values of subsequent studies tended to be lower. One study reported an allelic OR of 5.10 and a genotypic OR of 19.40 (based on an autosomal recessive mode of inheritance), and a more recent study reported an OR of 8.5 (Vt/Vt versus Wt/Wt) (36, 52). Our results are similar to the subsequent studies with allelic and genotypic ORs of 3.59 and 13.09, respectively, and an OR of 11.25, if calculated as described in the study of Stern et al. (52). For MYBPC3:c.220G > A [A74T], the initial study reported a genotypic OR (based on an autosomal recessive mode of inheritance) of 7.6 (53), while a follow-up study found a value of 0.89 and the latter also resembles our allelic OR result of 2.56 (36). For MYBPC3:c.2453C > T [R818W], it is more difficult to compare the results with other replication studies: sample sizes were sometimes too low to assess association or data presentation did not allow exact OR calculation. Therefore, we were only able to calculate the range in which the allelic OR would have fallen in the original study and that is between 2.56 and 3.3, which means that the allelic OR of 35.82 observed in the current study indicates a larger effect (19, 36, 54). For TNNT2:c.95-108G > A, no OR was reported in the original publication, but based on the data, an allelic OR of 5 was found (22). In a subsequent study, the allelic OR and genotypic OR (based on an autosomal recessive mode of inheritance) were 1.2 and 2.43, which is similar to our results of 1.84 and 2.17, respectively (33). For MYH7:c.5647G > A [E1883K], no ORs were calculated as the variant was never observed, which is also according to expectations as it was postulated to be an extremely rare variant (21). Finally, for ALMS1:c.7384G > C [G2462R], only a relative risk of 13.6 and not an OR was reported in the original paper. However, this relative risk could not be reproduced based on the data that were provided. One subsequent study was not able to find an association between the variant and HCM development (45). This is in line with our result of an allelic and genotypic OR of 1.02 and 1.24 (autosomal recessive mode of inheritance).

Based on these results, three main observations can be made. The first is that the magnitude of the effect often drops between the first “discovery” study and subsequent validation studies. This is a finding that is regularly reported, underscoring the importance of conducting replication studies over time and across different geographic populations (55). The second observation is that our results are generally similar to the results obtained in other validation studies. This is important because this also implies that the subsequent classification was based on replicable results. The third and probably most important observation is that significant associations are reported for two variants only (MYBPC3:c.91G > C [A31P], MYBPC3:c.2453C > T [R818W]), while no association with HCM was reported for three others (MYBPC3:c.220G > A [A74T], TNNT2:c.95-108G > A, and ALMS1:c.7384G > C [G2462R]).

As there are no standardized criteria in veterinary genetics to evaluate whether there is sufficient evidence to consider a variant disease-causing, a decision on when the threshold is reached remains subjective. In human medicine, the ACMG guidelines were developed for that purpose, i.e., to help with the interpretation of newly discovered variants and to standardize their classification. Based on those criteria, only DNA tests of variants that fall in the category “pathogenic” should be used for clinical decisions (23). Variants that are “likely pathogenic” are indicative but should be combined with other evidence (23). For variants of unknown significance, the consensus is that they should not influence clinical decision-making but that the focus should be on resolving their unclear status (23).

Considering the high prevalence of HCM and as several variants were recently reported to be associated with HCM, we aimed to objectively evaluate the current evidence for the six variants published, based on the human ACMG guidelines, with one modification. This modification is linked to the use of the allelic frequency criteria that are tailored to the human situation. Even in humans, these cutoffs are sometimes too low, especially for highly prevalent diseases (23). As the population history of cats (and dogs) is characterized by population bottlenecks, the popular sire effect, and inbreeding, the situation is even more extreme in those species. Associated with that, the allelic frequencies of the disease-causing variants linked to these diseases are also higher. As a consequence, the criteria in the ACMG guidelines have reportedly been deemed too strict (34). For that reason, these criteria were not used in the initial classification but separately considered and compared to an alternative approach that takes disease prevalence into account.

Practically, based on the (modified) ACMG guidelines, two variants were classified as pathogenic, namely, MYBPC3:c.91G > C [A31P] and MYBPC3:c.2453C > T [R818W]. MYH7:c.5647G > A [E1883K] was classified as likely pathogenic. The other three (MYBPC3:c.220G > A [A74T], TNNT2:c.95-108G > A, and ALMS1:c.7384G > C [G2462R]) were all classified as variants of unknown significance. For four (MYBPC3:c.91G > C [A31P], MYBPC3:c.2453C > T [R818W], MYBPC3:c.220G > A [A74T], TNNT2:c.95-108G > A) of these six variants, this finding is a confirmation of earlier studies. In more detail, both the MYBPC3:c.91G > C [A31P] variant in the Maine Coon and the MYBPC3:c.2453C > T [R818W] in the Ragdoll have already been studied intensively, and while there is some disagreement on their actual mode of inheritance, their association with HCM is generally consistent (10, 36, 46–48, 52). For the other two (MYBPC3:c.220G > A [A74T], TNNT2:c.95-108G > A), several publications have discussed (and disproved) the initial association, and the MYBPC3:c.220G > A [A74T] variant was never published by the group that originally reported it (33, 36, 48).

As MYH7:c.5647G > A [E1883K] and ALMS1:c.7384G > C [G2462R] were only recently published, independent studies re-evaluating their association are limited. For the latter of the two (ALMS1:c.7384G > C [G2462R]), this study and another recently published study could not confirm the association with HCM (45). As too few other criteria supporting the classification are fulfilled, there is insufficient evidence to consider this variant as pathogenic or likely pathogenic; hence, we conclude that it is a variant of unknown significance. For the other variant (MYH7:c.5647G > A [E1883K]), the ACMG guidelines were already used for classification in the original publication. Recently, an independent study confirmed this low allelic frequency (47), and also in our study, this variant was absent from the entire cat population studied, albeit no domestic shorthairs were included here. In humans, the same variant has been described (ClinVar accession ID: VCV000014121.13), and based on a set of criteria that partially overlaps, the variant is there considered a variant of unknown significance. An in-depth comparison showed an agreement on the fulfillment of PP3 and PM2 (i.e., the computational evidence and that it is a rare variant) and that PM1 was not fulfilled (i.e., the location of the mutational hotspot ranged between amino acids 181 and 937, 51). The PP2 criterion (i.e., missense variants are a common mechanism) is not present anymore in the guidelines that resulted in the ClinVar classification, although they do state that it is “the predominant class of pathogenic alleles in MYH7” (42). They have an additional criterion that is fulfilled, namely, PS4, but with the weight set to support based on the observation that the allele occurred in five individuals consistent with the expected phenotype (i.e., these five individuals had HCM) (17, 56). In the original ACMG guidelines that were used here, this option is not available. The only disagreement was on PS3, a criterion that we considered fulfilled, and they did not. The decision to consider it fulfilled here was based on four papers: three of which did similar analyses and all showed a structural effect (38, 40, 56). The fourth paper did a movement analysis based on *C. elegans* and did not find an effect for any of the variants scrutinized, which makes it difficult to assess the suitability of the method itself as the other variants that were tested in that same paper were still considered to be pathogenic even with that result (41). Based on the overall agreement on whether criteria are fulfilled or not (i.e., there is only one that is debated, three that are agreed on, and two that have no direct match), the classification as “likely pathogenic” or “variant of unknown significance” seems linked to the system that is used. Among the authors, it did, however, raise various interesting questions and sparked debate: should the data of humans and animals be jointly considered as evidence? If the data are combined, there are six cases (five in humans, one in a cat), which lead to an increased weight of the PS4 criterion in the ClinVar classification and the fulfillment of the PS4 criterion in the ACMG guidelines applied in our study. However, this raises a question: should the PS4 criterion be applied species-specifically? To what extent can data be extrapolated across species either way? How should extremely rare variants be dealt with? While these questions are beyond the scope of the current study, they present interesting avenues for future studies. Overall, in the context of screening programs, we do not consider it necessary to include the MYH7:c.5647G > A [E1883K] variant as it is extremely rare (10).

Aside from the evaluation of these six variants with the guidelines, an additional goal was to compare the allelic frequency criteria of the ACMG and the alternative Tv-method. This can be done at two levels. The first level is checking whether the fulfillment of the criterion agrees with the classification based on all other criteria. For the Tv-method, this means that if the observed sample allele frequency is lower or equal to the cutoff, the frequency is consistent with the disease characteristics. If it is higher, that implies an inconsistency with theoretical expectations and that is a warning sign and indicates a benign role. For the ACMG, the cutoffs do not change with disease prevalence, penetrance, or genetic heterogeneity: an allelic frequency of >5% indicates a benign role, and (extremely) low allelic frequencies support a classification as pathogenic. Based on the Tv threshold, the allelic frequency of two out of two pathogenic, one out of the one classified as likely pathogenic, and two of the three variants classified as variants of unknown significance is correct. Based on the allelic frequency criteria of the ACMG guidelines, the allelic frequency of one out of two pathogenic, one out of one likely pathogenic, and three out of three variants classified as variants of unknown significance is correct; hence, the total agreement of the two methods at this level is identical (i.e., an agreement for five out of six variants).

The second level involves an evaluation of the effect on the final classification if the criterion would have been used as it is normally used. This is linked to the assigned weight of the criterion in the decision-making process. For the Tv threshold, the classification never changes: the two pathogenic variants remain pathogenic, the one likely pathogenic remains likely pathogenic, and the three others remain variants of unknown significance. For the ACMG guidelines, one pathogenic variant becomes a benign variant, and all three variants of unknown significance also become benign variants. Practically, this means that in the ACMG guidelines, this one criterion is enough to overrule all other evidence for 4/6 variants and that simply incorporating this criterion would change the classification drastically. Based on that result, the weight of the ACMG allelic frequency criteria seems to be too dominant in the decision-making process. However, based on the observation that even for highly prevalent diseases in humans, these cutoffs are too strict and considering that high disease prevalence (and the associated high allelic frequencies) occurs even far more often in domestic animals due to the breeding practices often used in those species, we doubt that the fixed ACMG cutoffs themselves are actually that suitable, in agreement with an earlier report (34). Broader than for HCM alone, as a wide range of variants has high allelic frequencies in cats (and dogs), if the BA1 stand-alone criterion would be applied to all of them, a large proportion would be declassified from (likely) pathogenic, irrespective of all the other information.

Interesting and similar to previous reports, the autosomal dominant mode of inheritance that was proposed for MYBPC3:c.91G > C [A31P] for Maine Coon cats in the original publication is less of an optimal fit to the data (Table 2). The autosomal recessive mode of inheritance seems a better fit to the data and is thus more likely (10, 36, 52). While an autosomal dominant mode of inheritance might make sense biochemically, statistically reports do not seem to find an increased risk for heterozygous cats, which is something one would expect if that is the correct mode of inheritance (10, 36). The difference between autosomal recessive and autosomal dominant might at some point become a semantic discussion (36); however, when informing owners on the risk, they must realize that the risk of developing HCM does not seem to be increased in heterozygous cats based on our results. Taking that into account, altering breeding advice for that mutation to an approach similar to what would be done for autosomal recessive diseases, especially when genetic diversity is low(er), might also be considered (24, 25). Noteworthy, for MYBPC3:c.2453C > T [R818W], the distribution of genotypes across cases and controls made it impossible to discriminate an autosomal recessive or dominant mode of inheritance. Hence, there is no evidence against the originally proposed autosomal dominant mode of inheritance (19).

### 4.1 Limitations of the study

The samples collected in this study were derived from two main sources. The added value of the samples of the commercial laboratories is clear: large sample sizes make by chance deviations less probable and increase the probability to detect rare events. There are, however, two downsides: breeds are reported by the owners themselves for these samples, which makes it of course impossible to check whether breed was correctly specified. Furthermore, while the potential presence of duplicate samples from batch one could be ruled out, an absence of overlap between the samples from the commercial laboratories could not entirely be guaranteed. Both might have had an influence on the allelic frequencies of the MYBPC3:c.91G > C [A31P] and the MYBPC3:c.2453C > T [R818W] variants. However, as none of the results related to allelic frequencies was unexpected, we deem it unlikely that this has influenced the results drastically. A second limitation is linked to the international collaboration that has increased the sample size in this study: this might have led to variation in phenotypical classification. In more detail, several veterinarians were involved, and it has been shown that the interpretation of cardiac ultrasound measurements can differ between observers (57). However, an attempt at standardization was made by using internationally accepted consensus guidelines for echocardiographic phenotypic classification (29). Furthermore, those cats for which the phenotype was not entirely clear (=the equivocal cats) were excluded from the association part of the study. As the median age of the healthy control group was very high, this makes it far less likely to have a substantial proportion of false negative cats (i.e., cats that still will develop HCM, but did not have it at the time of the screening) in the control group. Based on all these combinations, the probability of misclassification was reduced as much as possible. A third limitation is linked to the absence of domestic short- and longhair cats from the study population. It can be argued that this absence might have resulted in missing the MYH7:c.5647G > A [E1883K] variant. We deem this unlikely, however: both in the original publication and in a recently published one, a large set of domestic shorthairs were included, leading to a combined sample size of 196 cats (21, 47). As the variant was never found in either of the publications, it seems very rare and the current data only support this with the observed absence in a large dataset of several breeds, including one breed (=the Sphynx) that had not been tested yet. A final limitation is that while we considered several modes of inheritance, these are only a subset of all the modes that are possible, and this subset might not fully capture the intricacies of HCM’s genetic landscape.

## 5 Conclusion

Based on the separate phases in this study, the evaluation with the ACMG guidelines is in line with previous research, two variants were found to be pathogenic, and one variant was classified as likely pathogenic. However, practically we recommend limiting routine genetic testing to two variants in two breeds, i.e., the MYBPC3:c.91G > C [A31P] in Maine Coon and the MYBPC3:c.2453C > T [R818W] variant in Ragdolls (10). While their (homozygous) presence increases the risk of developing HCM in those two breeds, they are virtually absent in other major breeds: the recommendation to specifically screen for these variants in the breeds in which they were originally described, but not other breeds, thus remains (36, 58, 59). While the MYH7:c.5647G > A [E1883K] variant is categorized as likely pathogenic, its rare occurrence renders routine testing for this variant unnecessary (10). The other variants lack evidence for disease causation, leading to a consensus against screening for these variants in any breed (36, 58, 59). Overall, the human ACMG guidelines are a good starting point, but one modification has already been shown to perform at least as well as the traditional allelic frequency criteria. Furthermore, interesting questions were raised during the application of these guidelines. As such, a thorough and evidence-based evaluation (and potential modification) of the ACMG guidelines would be an interesting next step to optimize their applicability in veterinary medicine.

## Data availability statement

The original contributions presented in the study are included in the article/Supplementary material, further inquiries can be directed to the corresponding authors.

## Ethics statement

All included cats had informed owner consent. Samples were non-invasive buccal swabs or whole blood on EDTA. In some cats, blood was obtained as part of routine clinical procedures for diagnostic purposes, at the request and with the consent of the owner. As these samples were from client-owned cats for which no harmful invasive procedures were performed, there was no animal experimentation according to the legal definition in Europe (Subject 5f of Article1, Chapter I of the Directive 2010/63/UE of the European Parliament and of the Council). A subset of the blood samples was collected as part of a biobanking initiative, approved by the Ethics Committee of the Faculties of Veterinary Medicine and Bioscience Engineering at Ghent University (EC2017-86). Samples from Sweden were collected in agreement with the ethical committee of the Swedish Board of Agriculture [No. C2/12 (2012-02-24),C12/15 (2015-02-27), and Dnr 5.8.18–04682/2020]. Samples sourced from the Cornell Veterinary Biobank were collected under Institutional Animal Care and Use Committee (IACUC) approved animal protocol #2005–0151. The studies were conducted in accordance with the local legislation and institutional requirements. Written informed consent was obtained from the owners for the participation of their animals in this study.

## Author contributions

FB: Conceptualization, Data curation, Formal analysis, Resources, Writing – original draft, Writing – review & editing. MA: Conceptualization, Resources, Writing – review & editing. HA: Conceptualization, Resources, Writing – review & editing. TD: Conceptualization, Resources, Writing – review & editing. PF: Conceptualization, Resources, Writing – review & editing. PRF: Conceptualization, Resources, Writing – review & editing. JJH: Conceptualization, Resources, Writing – review & editing. JH: Conceptualization, Resources, Writing – review & editing. SD: Conceptualization, Resources, Writing – review & editing. MK: Conceptualization, Resources, Writing – review & editing. FS: Conceptualization, Resources, Writing – review & editing. IL: Conceptualization, Resources, Writing – review & editing. LL: Conceptualization, Resources, Writing – review & editing. ML: Conceptualization, Resources, Writing – review & editing. ÅO: Conceptualization, Resources, Writing – review & editing. LP: Conceptualization, Resources, Writing – review & editing. CC: Conceptualization, Resources, Writing – review & editing. PS: Conceptualization, Resources, Writing – review & editing. MT: Conceptualization, Resources, Writing – review & editing. BB: Conceptualization, Data curation, Formal analysis, Resources, Supervision, Writing – original draft, Writing – review & editing.

## Footnotes

^1^ http://genetics.bwh.harvard.edu/pph2/

^2^ https://sift.bii.a-star.edu.sg/www/SIFT_seq_submit2.html

^3^ http://mutpred2.mutdb.org/#qform

^4^ http://wangcomputing.com/assp/

^5^ http://hollywood.mit.edu/GENSCAN.html

^6^ https://services.healthtech.dtu.dk/service.php?NetGene2-2.42
