# Corrigendum: Classification of feline hypertrophic cardiomyopathy-associated gene variants according to the American College of Medical Genetics and Genomics guidelines

**DOI:** 10.3389/fvets.2024.1458433

**Published:** 2024-08-12

**Authors:** Fréderique Boeykens, Marie Abitbol, Heidi Anderson, Tanushri Dargar, Paolo Ferrari, Philip R. Fox, Jessica J. Hayward, Jens Häggström, Stephen Davison, Mark D. Kittleson, Frank van Steenbeek, Ingrid Ljungvall, Leslie A. Lyons, Maria Longeri, Åsa Ohlsson, Luc Peelman, Caroline Dufaure de Citres, Pascale Smets, Maria Elena Turba, Bart J. G. Broeckx

**Affiliations:** ^1^Laboratory Animal Genetics, Department of Veterinary and Biosciences, Faculty of Veterinary Medicine, Ghent University, Merelbeke, Belgium; ^2^Univ Lyon, VetAgro Sup, Marcy-l'Etoile, France & Institut NeuroMyoGène INMG-PNMG, CNRS UMR5261, INSERM U1315, Faculté de Médicine, Rockefeller, Université Claude Bernard Lyon 1, Lyon, France; ^3^Wisdom Panel, Mars Petcare Science & Diagnostics, Helsinki, Finland; ^4^Osservatorio Veterinario Italiano Cardiopatie, Azzano San Paolo, Italy; ^5^Bis Clinica Veterinaria Orobica Anicura, Bergamo, Italy; ^6^The Animal Medical Center, New York, NY, United States; ^7^Department of Biomedical Sciences and Cornell Veterinary Biobank, College of Veterinary Medicine, Cornell University, Ithaca, NY, United States; ^8^Department of Clinical Sciences, Faculty of Veterinary Medicine and Animal Science, Swedish University of Agricultural Sciences, Uppsala, Sweden; ^9^Wisdom Panel, Mars Petcare Science & Diagnostics, Leicestershire, United Kingdom; ^10^Veterinary Information Network and School of Veterinary Medicine and Epidemiology, University of California, Davis, Davis, CA, United States; ^11^Department of Clinical Sciences, Faculty of Veterinary Medicine, Utrecht University, Utrecht, Netherlands; ^12^Department of Veterinary Medicine and Surgery, College of Veterinary Medicine, University of Missouri, Columbia, MO, United States; ^13^Department of Veterinary Medicine and Animal Sciences, University of Milan, Lodi, Italy; ^14^Department of Animal Breeding and Genetics, Faculty of Veterinary Medicine and Animal Science, Swedish University of Agricultural Sciences, Uppsala, Sweden; ^15^Antagene, La Tour-de-Salvagny, France; ^16^Small Animal Department, Ghent University, Merelbeke, Belgium; ^17^Genefast srl, Forlì, Italy

**Keywords:** cardiac disease, feline genetics, variant classification, ACMG guidelines, genetic diversity

In the published article, there was an error: the wording in the phenotype subsection was imprecise.

A correction has been made to **2 Materials and Methods, 2.5 Phenotyping**. These sentences previously stated:

“A left ventricular wall thickness during end-diastole below 5 mm is regarded as within the physiological norm, and a thickness equal to or exceeding 6 mm is indicative of hypertrophy. It is noteworthy that reference ranges, incorporating considerations of body weight, were applied as an additional parameter (1). Cats with a wall thickness measurement between 5.5 and 5.9 mm outside the body weight-based reference interval, and with normal left atrial size, were classified as equivocal.”

The corrected sentences appear below:

“In the realm of feline cardiology, there is no universally accepted standard for determining the normal thickness of the left ventricular wall during diastole. It is overly simplistic to assume that a single numerical value can reliably distinguish between a healthy left ventricular wall and one affected by hypertrophy across all cats. The thickness of the left ventricular wall increases nonlinearly with body weight and is influenced by physiological factors such as hydration level and heart rate. Conditions like hyperthyroidism and systemic hypertension, which are common in older cats, can further impact left ventricular wall thickness, and the presence of these conditions were examined in cats, if suspected, before inclusion.

Nevertheless, for most cats that are not hyperthyroid or hypertensive, of average size and with a normal body condition (weighing between 3.5–5 kg), a left ventricular diastolic wall thickness of 5 mm or less is typically considered within the normal range, while a thickness of 6 mm or more suggests concentric hypertrophy. Cats with a left ventricular wall thickness falling between 5 and 6 mm were termed equivocal and were excluded from analysis. Cats outside this weight range were managed on a case to case basis with the aid of previously published 95% prediction intervals (1).”

The authors apologize for this error and state that this does not change the scientific conclusions of the article in any way. The original article has been updated.

