# Supplementary material for: Classification of feline hypertrophic cardiomyopathy-associated gene variants according to the American College of Medical Genetics and Genomics guidelines
Source: Front Vet Sci. 2024 Feb 2;11:1327081. doi: 10.3389/fvets.2024.1327081 (PMC10873919; doi:10.3389/fvets.2024.1327081)
Supplement: Supplementary file 1 [file Data_Sheet_1.docx]

Supplementary Material

## Supplementary Tables

| **Supplementary Table 1: Overview of Feline Samples Used in the Study**. The table details the number of samples obtained from the Wisdom Panel (Mars) commercial laboratory that were already included in an earlier publication, the total number of samples published here, and the proportion of Wisdom Panel samples relative to this total. | | | |
| --- | --- | --- | --- |
| Breed | # samples Wisdom | Total # samples | Proportion (%) |
| British Short- and Longhair | 390 | 878 | 44.42 |
| Ragdoll | 1115 | 4533 | 24.60 |
| Sphynx | 547 | 1159 | 47.20 |
| Maine Coon | 2121 | 14025 | 15.12 |
| Devon Rex | 447 | 956 | 46.76 |

| **Supplementary Table 2:** Power analysis with epiR R-package to determine the minimal number of cases (N_case_) and controls (N_control_) needed to obtain a significant odds ratio (OR) with a power of 80%. A disease-prevalence of 15% is assumed in the group without the disease-causing allele, and a balanced number of affected and healthy cats was assumed. While the calculation was based on allelic ORs, N details the number of individuals (and represents the number of alleles divided by two). | | |
| --- | --- | --- |
|  | Allelic OR | N_case_ - N_control_ |
| MYBPC3:c.91G>C [A31P] | 62.14 | 3 – 3 |
| MYBPC3:c.220G>A [A74T] | 7.6 | 10 – 10 |
| MYBPC3:c.2453C>T [R818W] | 1131.26 | 2 – 2 |
| TNNT2:c.95-108G>A | 5 | 16 – 16 |
| ALMS1:c.7384G>C [G2462R] | 13.6 | 6 – 6 |
| MYH7:c.5647G>A [E1883K] | 2403 | 2 – 2 |

| **Supplementary Table 3:** Overview of genotyping methods for the six HCM-associated variants. | | | | | | |
| --- | --- | --- | --- | --- | --- | --- |
| MYBPC3:c.91G>C [A31P] and MYBPC3:c.220G>A [A74T] | | | | | | |
| Forward primer | | | | TCAGCCTTCAGCAAGAAGCCA | | |
| Reverse primer | | | | GCTGCCCCAGAAGCTCAAAC | | |
| Product length | | | | 311 | | |
| PCR Mix | | | | PCR program | | |
| 4.7  0.1  2  1  1  0.2  1 | μl Water  μl TEMPase Hot Start DNA polymerase  μl Template DNA  μl GC-rich  μl TEMPase 10x buffer (+ 20 mM MgCl_2_)  μl dNTPs (10 mM each)  μl Primers (5 μM each) | | | 95.0 °C  95.0 °C  62.0 °C  72.0 °C  72.0 °C  10.0 °C | 14:30  00:30  00:30 30x  01:00  10:00  HOLD | |
| Sequencing mix | | | | Sequencing program | | |
| 2  0.5  1.5  1  3 | μl 5x Sequencing buffer  μl BigDye Terminator RR-mix  μl Primer (forward or reverse)  μl GC-rich  μl Water | | | 95.0 °C  60.0 °C  65.0 °C  10.0 °C | 02:00  00:20  00:10 30x  HOLD | |
| Alternative | | | | | |  |
| Forward primer | | | AGTCTCAGCCTTCAGCAAGAAGCC | | |  |
| Reverse primer | | | GGTCAAACTTGACCTTGGAGGAGCC | | |  |
| Product length | | | 250 | | |  |
| Other adjustments | | | Platinum Taq DNA polymerase (Invitrogen)  Initial denaturation: 02:00  Annealing temperature: 60.0°C | | |  |
| Alternative II | | |  | | |  |
| Forward primer | | | TCTCATAGAGCCACTGAAGCATTA | | |  |
| Reverse primer | | | CTCAGAACTTTCCCTACTTCCACA | | |  |
| Annealing temperature | | | 59°C | | |  |
|  | | |  | | |  |
| TNNT2:c.95-108G>A | | | | | | |
| Forward primer | | | | CCTCACCTTCAGCCTCTTCT | | |
| Reverse primer | | | | CGCACCCTAACACACTCCTA | | |
| Product length | | | | 539 | | |
| PCR Mix | | | | PCR program | | |
| 4.7  0.1  2  1  1  0.2  1 | μl Water  μl TEMPase Hot Start DNA polymerase  μl Template DNA  μl GC-rich  μl TEMPase 10x buffer (+ 20 mM MgCl_2_)  μl dNTPs (10 mM each)  μl Primers (5 μM each) | | | 95.0 °C  95.0 °C  62.0 °C  72.0 °C  72.0 °C  10.0 °C | 14:30  00:30  00:30 30x  01:00  10:00  HOLD | |
| Sequencing mix | | | | Sequencing program | | |
| 2  0.5  1.5  1  3 | μl 5x Sequencing buffer  μl BigDye Terminator RR-mix  μl Primer (forward)  μl GC-rich  μl Water | | | 95.0 °C  60.0 °C  65.0 °C  10.0 °C | 02:00  00:20  00:10 30x  HOLD | |
| MYBPC3:c.2453C>T [R818W] | | | | | | |
| Forward primer | | | | GACCAGAGCTCCTGTC | | |
| Reverse primer | | | | GCGTAGACTCGCATCTCGTA | | |
| Product length | | | | 121 | | |
| Probe wild type | | | | FAM-TTCAGCCGCATCCACC-BHQ1 | | |
| Probe variant type | | | | HEX-AGTTCAGCCACATCCACC-BHQ1 | | |
| PCR Mix | | | | PCR program | | |
| 4.7  0.1  2  1  1  0.2  1 | μl Water  μl TEMPase Hot Start DNA polymerase  μl Template DNA  μl GC-rich  μl TEMPase 10x buffer (+ 20 mM MgCl_2_)  μl dNTPs (10 mM each)  μl Primers (5 μM each) | | | 95.0 °C  95.0 °C  60.0 °C  72.0 °C  72.0 °C  10.0 °C | 14:30  00:30  00:30 30x  01:00  10:00  HOLD | |
| TaqMan assay: make sure to dilute DNA-sample 1:100 | | | | | | |
| TaqMan Assay | | | | qPCR program | | |
| 4.0  1  1  0.5  0.2  0.2  0.1  1  2 | μl Water  μl 10x Key buffer  μl Primers (5 μM each)  μl WT probe (FAM) (10 μM)  μl VT probe (HEX) (10 μM)  μl dNTPs (10 μM each)  μl TEMPase polymerase (5U/μl)  μl GC-rich  μl DNA (1:100 diluted) | | | 95.0 °C  95.0 °C  57.0 °C  Plate read | 14:40  00:20  00:40 39x | |
| Alternative | | | | | |  |
| Forward primer | | | | CAGCAATGTGGGTGAGGAC | |  |
| Reverse primer | | | | CTGACCAGGGAGGGTGTG | |  |
| Product length | | | | 396 | |  |
| Other adjustments | | | | Platinum Taq DNA Polymerase (Invitrogen)  Initial denaturation 02:00  Annealing temperature 60°C | |  |
| Alternative II | | | |  | |  |
| Forward primer | | | | CCCAAGATCAGCAATGTGGG | |  |
| Reverse primer | | | | GGACCCGGATGTAAATGCCT | |  |
| Annealing temperature | | | | 58°C | |  |
| MYH7:c.5647G>A [E1883K] | | | | | | |
| Forward primer | | | | GGTAACGACCACGGCGGGAGA | | |
| Reverse primer | | | | CGCTCCTCTGCCTCATCCAGCTC | | |
| PCR Mix | | | | PCR program | | |
| 4.7  0.1  2  1  1  0.2  1 | μl Water  μl TEMPase Hot Start DNA polymerase  μl Template DNA  μl GC-rich  μl Tempase 10x buffer (+ 20 mM MgCl_2_)  μl dNTPs (10 mM each)  μl Primers (5 μM each) | | | 95.0 °C  95.0 °C  62.0 °C  72.0 °C  72.0 °C  10.0 °C | 14:30  00:30  00:30 30x  01:00  10:00  HOLD | |
| RFLP-test with BseRI enzyme | | | |  | | |
| 1  1.5  0.5 | μl Cutsmart buffer  μl BseRI (7.5 U)  μl Water | | | 37 °C  3 μl mix with PCR product | | |
| Alternative | | | | | |  |
| Forward primer | | CCCTCCTCACTCCTAACCCT | | | |  |
| Reverse primer | | TGACATGCGGTGACTAGTGG | | | |  |
| Annealing temperature | | 58°C | | | |  |
| ALMS1:c.7384G>C [G2462R] | | | | | | |
| Forward primer | | | | TCCCCTTCTGATCACACTGC | | |
| Reverse primer | | | | CCCCTCAGAAAGATAATGCAGG | | |
| Product length | | | | 306 | | |
| PCR Mix | | | | PCR program | | |
| 4.7  0.1  2  1  1  0.2  1 | μl Water  μl TEMPase Hot Start DNA polymerase  μl Template DNA  μl GC-rich  μl TEMPase 10x buffer (+ 20 mM MgCl_2_)  μl dNTPs (10 mM each)  μl Primers (5 μM each) | | | 95.0 °C  95.0 °C  62.0 °C  72.0 °C  72.0 °C  10.0 °C | 14:30  00:30  00:30 30x  01:00  10:00  HOLD | |
| Sequencing mix | | | | Sequencing program | | |
| 2  0.5  1.5  1  3 | μl 5x Sequencing buffer  μl BigDye Terminator RR-mix  μl Primer (forward)  μl GC-rich  μl Water | | | 95.0 °C  60.0 °C  65.0 °C  10.0 °C | 02:00  00:20  00:10 30x  HOLD | |

| **Supplementary table 4:** A concise overview of the criteria for classifying pathogenic and benign variants. These criteria are summarized from (Richards et al. 2015). Only the criteria used in the classification process are mentioned. Each criterion has a unique identifier, which is a combination of whether it supports a pathogenic or benign classification (P or B), followed by the weight assigned to that criterion (from high to low: stand-alone (A), very strong (VS), strong (S), moderate (M) or supporting (P)) and finally a number. This number has no function besides making it more easy to refer to that specific criterion. |
| --- |
| *Criteria that support a classification as pathogenic* |
| **Very Strong Evidence (PVS1):** null variants in genes where loss of function is a known disease mechanism, with caveats for certain gene types or variant locations. |
| **Strong Evidence (PS1-PS4):** covers scenarios like identical amino acid changes to known pathogenic variants (PS1), functional studies supporting a damaging effect (PS3), and significantly increased prevalence in affected individuals relative to controls (PS4), amongst others. |
| **Moderate Evidence (PM1-PM6):** involves criteria like location in mutational hot spots (PM1), absence form control populations (PM2), a novel missense change at a position where a different missense change is known to be pathogenic (PM5), amongst others.. |
| **Supporting Evidence (PP1-PP5):** encompasses criteria such as the knowledge that a missense variant is a common disease mechanism (PP2) and computational evidence of deleterious effects (PP3), amongst others. |
| *Criteria that support a classification as benign* |
| **Stand-alone Evidence (BA1):** a variant with an allele frequency above 5% in major genomic databases like the Exome Sequencing Project, 1000 Genomes, or ExAc is considered benign. |
| **Strong Evidence (BS1-BS4):** this includes cases where allele frequency exceeds disorder expectations (BS1), amongst others. |
| **Supporting Evidence (BP1-BP7)**: none of the criteria applicable in this study. |

| **Supplementary Table 5:** Calculation of the alternative allele frequency thresholds Tv. | | | | | | | | | |
| --- | --- | --- | --- | --- | --- | --- | --- | --- | --- |
| Variant | Database size | Database type | Mode of inheritance | Number of loci^1^ | HCM Prevalence | Detectance | Penetrance | Probability | Calculated threshold Tv |
| Maine Coon | | | | | | | | | |
| MYBPC3:c.91G>C [A31P] | 14025 | a | AD | 1 | 30% | 100% | 100% | 0.95 | 0.17 |
| MYBPC3:c.220G>A [A74T] | 95 | a | AR | 2 | 30% | ≈20% | 50% | 0.95 | 0.41 |
| TNNT2:c.95-108G>A | 97 | a | AR | 3 | 30% | 16% | 100% | 0.95 | 0.27 |
| Ragdoll | | | | | | | | | |
| MYBPC3:c.2453C>T [R818W] | 4754 | a | AD | 1 | 20% | 100% | 100% | 0.95 | 0.11 |
| Sphynx | | | | | | | | | |
| ALMS1:c.7384G>C [G2462R] | 107 | a | AD | 1+1 | 20% | 87% | 77% | 0.95 | 0.15 |
| Domestic Shorthair | | | | | | | | | |
| MYH7:c.5647G>A [E1883K] | 277^2^ | a | AD | 1+1 | 15% | 0.18% | 100% | 0.95 | 0.02 |
| ^1^ the number of loci before the plus sign is the number of loci that were published at that time for that specific breed, the “+1” indicates that at the time of publication, it was already recognized that a certain proportion of cats with HCM was unexplained by those loci, respectively. ^2^no Domestic Shorthairs were included in the database. As this is a rare mutation according to the original study, the proportion of HCM caused by that variant was set to be at most once in the entire database, where the database size reflects the total number of animals successfully tested for this variant. | | | | | | | | | |

| **Supplementary Table 6:** Overview of test results per year for the MYBPC3:c.2455C>T and MYBPC3:c.91G>C variants that were genotyped in Ragdolls and Maine Coons, respectively. The total (n) number of samples tested per year are shown, together with the homozygous wildtype (Wt/Wt), heterozygotes (Wt/Vt) and the homozygous (Vt/Vt) variant individuals, respectively. Furthermore, the allelic frequency (q) was calculated for each year. | | | | | |
| --- | --- | --- | --- | --- | --- |
| **Year** | **Total (n)** | **Wt/Wt** | **Wt/Vt** | **Vt/Vt** | **q (%)** |
| MYBPC3:c.2453C>T [R818W] (Ragdoll) | | | | | |
| 2008 | 32 | 22 | 10 | 0 | 15.63 |
| 2009 | 120 | 84 | 34 | 2 | 15.83 |
| 2010 | 142 | 103 | 39 | 0 | 13.73 |
| 2011 | 119 | 108 | 11 | 0 | 4.62 |
| 2012 | 123 | 112 | 11 | 0 | 4.47 |
| 2013 | 128 | 104 | 24 | 0 | 9.38 |
| 2014 | 98 | 87 | 11 | 0 | 5.61 |
| 2015 | 122 | 116 | 6 | 0 | 2.46 |
| 2016 | 107 | 101 | 6 | 0 | 2.80 |
| 2017 | 141 | 140 | 1 | 0 | 0.35 |
| 2018 | 108 | 106 | 2 | 0 | 0.93 |
| 2019 | 134 | 133 | 1 | 0 | 0.37 |
| 2020 | 159 | 159 | 0 | 0 | 0.0 |
| 2021 | 242 | 242 | 0 | 0 | 0.0 |
| MYBPC3:c.91G>C [A31P] (Maine Coon) | | | | | |
| 2008 | 261 | 164 | 92 | 5 | 19.54 |
| 2009 | 579 | 393 | 180 | 6 | 16.58 |
| 2010 | 563 | 406 | 151 | 6 | 14.48 |
| 2011 | 571 | 444 | 122 | 5 | 11.56 |
| 2012 | 758 | 636 | 118 | 4 | 8.31 |
| 2013 | 649 | 559 | 88 | 2 | 7.09 |
| 2014 | 635 | 568 | 65 | 2 | 5.43 |
| 2015 | 644 | 589 | 53 | 2 | 4.43 |
| 2016 | 552 | 514 | 38 | 0 | 3.44 |
| 2017 | 475 | 448 | 27 | 0 | 2.84 |
| 2018 | 482 | 467 | 15 | 0 | 1.56 |
| 2019 | 610 | 589 | 20 | 1 | 1.80 |
| 2020 | 860 | 828 | 31 | 1 | 1.92 |
| 2021 | 1209 | 1186 | 23 | 0 | 0.95 |

| **Supplementary Table 7:** Overview of the allelic and genotypic odds ratios (ORs) for the 3 variants that were not significant (MYBPC3:c.220G>A, TNNT2:c.95-108G>A and ALMS1:c.7384G>C) in the initial analysis and for which the analysis thus was repeated across all breeds studied. To be included in this analysis, a cat had to be homozygous for the wildtype allele for all variants except for the one evaluated. The total sample size is reported, as well as the number of cats per breeds that led to this sample size. The genotypic OR that corresponds with the mode of inheritance proposed in the original paper, is underlined. n: total number of cats; Wt/Wt: homozygous wild-type; Wt/Vt: heterozygous, Vt/Vt: homozygous variant; IP: inheritance pattern; OR: odds ratio; CI: 95% confidence interval; A: Allelic OR; R: autosomal recessive genotypic OR; D: autosomal dominant genotypic OR. | | | | | | | | | | |
| --- | --- | --- | --- | --- | --- | --- | --- | --- | --- | --- |
| Variant | n | Case | | | Control | | | IP | OR | CI |
|  |  | Wt/Wt | Wt/Vt | Vt/Vt | Wt/Wt | Wt/Vt | Vt/Vt |  |  |  |
| **MYBPC3:c.220G>A [A74T]** | **84** | 14 | 26 | 6 | 17 | 18 | 3 | A | 1.52 | [0.80-2.88] |
| British | 26 |  |  |  |  |  |  | R | 1.75 | [0.41-7.52] |
| Devon Rex | 12 |  |  |  |  |  |  | D | 1.85 | [0.76-4.53] |
| Maine Coon | 23 |  |  |  |  |  |  |  |  |  |
| Ragdoll | 7 |  |  |  |  |  |  |  |  |  |
| Sphynx | 16 |  |  |  |  |  |  |  |  |  |
| **TNNT2:c.95-108G>A** | **39** | 14 | 2 | 1 | 17 | 4 | 1 | A | .84 | [0.22-3.27] |
| British | 9 |  |  |  |  |  |  |  |  |  |
| Devon Rex | 0 |  |  |  |  |  |  | R | 1.31 | [0.08-22.63] |
| Maine Coon | 24 |  |  |  |  |  |  |  |  |  |
| Ragdoll | 4 |  |  |  |  |  |  | D | 0.73 | [0.15-3.60] |
| Sphynx | 2 |  |  |  |  |  |  |  |  |  |
| **ALMS1:c.7384G>C [G2462R]** | **38** | 14 | 3 | 1 | 17 | 3 | 0 | A | 1.99 | [0.44-8.99] |
| British | 9 |  |  |  |  |  |  |  |  |  |
| Devon Rex | 2 |  |  |  |  |  |  | R | 3.51 | [0.13-91.88] |
| Maine Coon | 17 |  |  |  |  |  |  |  |  |  |
| Ragdoll | 3 |  |  |  |  |  |  | D | 1.62 | [0.31-8.48] |
| Sphynx | 7 |  |  |  |  |  |  |  |  |  |
